# *In silico* Hierarchical Clustering of Neuronal Populations in the Rat Ventral Tegmental Area Based on Extracellular Electrophysiological Properties

**DOI:** 10.3389/fncir.2020.00051

**Published:** 2020-08-13

**Authors:** Mathieu Di Miceli, Zoé Husson, Philippe Ruel, Sophie Layé, Daniela Cota, Xavier Fioramonti, Clémentine Bosch-Bouju, Benjamin Gronier

**Affiliations:** ^1^Pharmacology and Neuroscience Research Group, Leicester School of Pharmacy, De Montfort University, Leicester, United Kingdom; ^2^Laboratoire NutriNeuro, UMR INRAE 1286, Université de Bordeaux, Bordeaux, France; ^3^INSERM, Neurocentre Magendie, Physiopathologie de la Plasticité Neuronale, University of Bordeaux, Bordeaux, France; ^4^IGF, Université de Montpellier, CNRS, INSERM, Montpellier, France; ^5^Département de Mathématiques, Lycée Joffre, Académie de Montpellier, Montpellier, France

**Keywords:** electrophysiology, VTA, hierarchical clustering, dopaminergic neurons, neurophysiology

## Abstract

The ventral tegmental area (VTA) is a heterogeneous brain region, containing different neuronal populations. During *in vivo* recordings, electrophysiological characteristics are classically used to distinguish the different populations. However, the VTA is also considered as a region harboring neurons with heterogeneous properties. In the present study, we aimed to classify VTA neurons using *in silico* approaches, in an attempt to determine if homogeneous populations could be extracted. Thus, we recorded 291 VTA neurons during *in vivo* extracellular recordings in anesthetized rats. Initially, 22 neurons with high firing rates (>10 Hz) and short-lasting action potentials (AP) were considered as a separate subpopulation, in light of previous studies. To segregate the remaining 269 neurons, presumably dopaminergic (DA), we performed *in silico* analyses, using a combination of different electrophysiological parameters. These parameters included: (1) firing rate; (2) firing rate coefficient of variation (CV); (3) percentage of spikes in a burst; (4) AP duration; (5) Δt_1_ duration (i.e., time from initiation of depolarization until end of repolarization); and (6) presence of a notched AP waveform. Unsupervised hierarchical clustering revealed two neuronal populations that differed in their bursting activities. The largest population presented low bursting activities (<17.5% of total spikes in burst), while the remaining neurons presented higher bursting activities (>17.5%). Within non-high-firing neurons, a large heterogeneity was noted concerning AP characteristics. In conclusion, this analysis based on conventional electrophysiological criteria clustered two subpopulations of putative DA VTA neurons that are distinguishable by their firing patterns (firing rates and bursting activities) but not their AP properties.

## Introduction

Mesolimbic dopaminergic (DA) structures control motivation and reward (Taylor et al., 2016). Amongst midbrain DA structures, the ventral tegmental area (VTA) has been extensively studied. Outputs from the VTA include the ventral striatum and the anterior cortex (Beier et al., 2015). Recently, several studies have highlighted some heterogeneity amongst VTA neurons, especially concerning their input/output circuitry (Morales and Margolis, 2017; Poulin et al., 2018), their responses to DA pharmacology (Margolis et al., 2006, 2008), their electrophysiological characteristics (Luo et al., 2008) as well as their immunohistochemical signatures (Morales and Root, 2014; Yamaguchi et al., 2015). Therefore, the functions of the different neuronal subpopulations within VTA neurons are of interest.

DA neurons are extensively studied and, as such, electrophysiological identification of these neurons has been relying on several electrophysiological criteria. These include both firing patterns (such as firing rate, firing regularity and bursting activities) and action potential (AP) properties (width, shape, and presence of notched waveform), all summarized in a 2012 review by Ungless and Grace (2012). However, two studies identified putative DA neurons with electrophysiologically heterogeneous properties (Luo et al., 2008; Li et al., 2012).

In the present study, we investigated the heterogeneity of VTA neurons by combining all electrophysiological criteria in an attempt to classify neurons into different subpopulations. Here, we propose a simple and fast method for sorting non-high-firing neurons, presumably DA, that can be used during *in vivo* extracellular recordings, without requiring pharmacology or extensive analyses. First, high-firing neurons (>10 Hz) were considered as GABAergic neurons, following previous observations (Steffensen et al., 1998; Luo et al., 2008; Li et al., 2012). Since glutamatergic neurons account for 2–3% of all neurons within the VTA (Nair-Roberts et al., 2008), we considered the remaining non-high-firing neurons as DA neurons. Hierarchical clustering in these neurons revealed 2 subpopulations of DA neurons. Remarkably, these subpopulations differed by their firing patterns, but not their AP characteristics.

## Materials and Methods

### Ethical Approval

Experiments were performed in strict accordance with the recommendations in the Guide for the Care and Use of Laboratory Animals of the UK Home Office. All protocols were carried out under project and personal licenses issued by the UK Home Office under the UK Animals (Scientific Procedures) Act 1986 and were also approved by the Committee on the Ethics of Animal Experiments of De Montfort University (Protocol 60/4333). All experiments were performed under chloral hydrate anesthesia, administered at doses causing deep anesthesia (400 mg/kg as a starting dose) as previously described (Grace and Bunney, 1983, 1984; Ackerman et al., 1993; Marinelli and White, 2000; Zhang et al., 2008; Valenti et al., 2012; Marinelli and McCutcheon, 2014). Anesthesia was maintained by administering small intravenous boluses (~0.03 g/kg), typically every 8–10 min. Depth of anesthesia was confirmed by the absence of withdrawal following paw and tail pinches.

### Animals

Ninety-two adult male Sprague–Dawley rats (*P* > 56) were purchased from Charles River, UK. All animals were housed in groups of 2–4 per cage, maintained at 20–22°C with humidity rates above 40% under a 12:12 L/D cycle with lights ON at 07:00 h. Food and water were provided *ad libitum*. A cardboard tube was also placed in each cage to allow sheltering. Animals were allowed a 3-day acclimatization period after delivery. The 92 naive adult rats (250–450 g) included in the present study were also used in other studies with different objectives.

### *In vivo* Extracellular Single-Unit Electrophysiology

Animals were deeply anesthetized and the procedure started when the absence of withdrawal responses to paw and tail pinches was achieved. Animals were secured to a stereotaxic frame and maintained at 36–37°C. A catheter was inserted into the lateral tail vein to perform additional anesthetic administrations, if necessary, or to administer pharmacological agents. An incision was made across the top of the head and the edges of the skin drawn back to reveal the cranium. Bregma was marked and a hole was drilled through the bone at the coordinates of the VTA according to the atlas of Paxinos and Watson (Paxinos and Watson, 2007; anteroposterior −4.5 to −5.7 mm to Bregma, lateral 0.3–1.2 mm, 7.2–9.5 mm below the cortical surface). Electrodes were manufactured in house from borosilicate capillaries (1.5 mm, Harvard Apparatus Limited, UK) pulled on a PP-830 electrode puller (Narishige, Japan) and filled with an electrolyte solution (NaCl 147 mM, adjusted to pH 6 with NaOH). The tip of the electrode was broken down under a microscope to an external diameter of 1–1.5 μm. Typical resistance was in the range 4–8 MΩ. Outputs from the electrode were sent to a Neurolog AC pre-amplifier and amplifier (Digitimer, UK). Signals were filtered and sent to an audio amplifier, a Tektronix 2201 digital storage oscilloscope, and a 1,401 interface connected to a computer running Spike 2 v5.21 (CED, Cambridge, UK) for data capture and analysis. The descent of the electrode was carried out using a hydraulic micromanipulator (Narishige). At the end of the experimental procedures, animals were euthanized by intravenous chloral hydrate overdose. Brains were dissected out and cut using a cryostat (Leica) to visually confirm track locations within the VTA. Neurons located outside of the VTA were excluded from the present study. During some recordings, pharmacological agents (apomorphine 20–70 μg/kg, quinpirole 20 μg/kg, methylphenidate 2–4 mg/kg or eticlopride 0.2 mg/kg) were applied intravenously *via* the lateral vein tail catheter to test for firing rate inhibition through dopamine D_2_ autoreceptor negative feedback (Federici et al., 2005; Di Miceli et al., 2019).

### Data Analysis and Statistics

The mean basal firing activity was evaluated during at least 5 min, after achieving a stable firing rate. Burst activities were detected using a standard protocol (Grace and Bunney, 1984), where at least two spikes are occurring within an interval of 80 ms or less, followed by a silence period of at least 160 ms. For each neuron, burst activities are expressed as the percentage of all spikes occurring in bursts. Firing regularity was assessed by calculating coefficients of variation (CV) of the firing rates (standard deviation divided by mean). Both principal component analyses (PCA) and hierarchical cluster analyses were performed using R free software (R Core Team, 2013). PCA analysis was carried out to identify the parameters accounting for variability in our data set, as performed in another study (Mao et al., 2019), using the *prcomp* function in R (R Core Team, 2013; Jolliffe and Cadima, 2016) and drawn with the *ggbiplot* function (Vu, 2011). Raw data were analyzed using singular value decomposition of the scaled data matrix. More specifically, for each electrophysiological property, the dataset (matrix) was scaled from 0 to 1 to normalize (scaling) all values. Then, to adapt our scaled data matrix to multivariate analyses, such as PCA, the data was subsequently transformed using singular value decomposition, which performs linear transformations of a matrix. Such a process is a fundamental mathematical theorem that was explained previously (Golub and Van Loan, 1996). These transformations are applied directly with the aforementioned R functions. We used a total of six variables. These six variables were: (1) firing rate (i.e., the number of spikes generated in 1 s); (2) CV of firing rates (firing regularity index); (3) bursting activity (i.e., the percentage of all spikes included in bursting events); (4) AP duration (i.e., the total AP duration; (5) Δt_1_ duration (i.e., the time from initiation of depolarization until end of repolarization; as well as (6) notched AP waveforms (i.e., the presence or not of a notch). Then, we determined the optimal number of clusters within our data set by applying a silhouette analysis (Rousseeuw, 1987; Charrad et al., 2014; Mao et al., 2019), using the *Nbclust* function in R (R Core Team, 2013). Such an analysis is designed to determine optimal coherence amongst values. This method measures the quality of clustering by calculating the Euclidean distances between identified clusters and calculates the optimal number of clusters to achieve ideal separation between all values, based on an array computation of 30 indices (Charrad et al., 2014). Finally, raw data was plotted using hierarchical analysis with Ward’s method (Ward, 1963), based on the ideal cluster number found following the silhouette analysis. The resulting cluster dendrogram was generated using *eclust* and *fviz_dend* functions (Kassambara and Mundt, 2017). Results are expressed as the mean ± standard error of the mean (SEM). On box-plots (box-and-whiskers), means are represented by a cross (+), together with the 5–95% percentile range. Outliers out of the 5–95% percentile were plotted individually but were not excluded from all analyses. For comparison between three groups, the Kruskal–Wallis one-way analysis of variance (ANOVA) tests were performed, followed by Dunn’s *post hoc* tests. For contingency analyses, chi-square tests were used. For all tests, statistical significance was considered if *p* < 0.05. All statistical tests were performed with GraphPad Prism 7.0 (GraphPad Software Inc., San Diego, CA, USA). All graphs were drawn using GraphPad, except silhouettes, PCA and dendrograms, which were drawn using R (R Core Team, 2013). Graphs were designed using colours optimized for the color-blind (Cox, 2015).

## Results

### Dataset Characteristics

**Figure 1:**
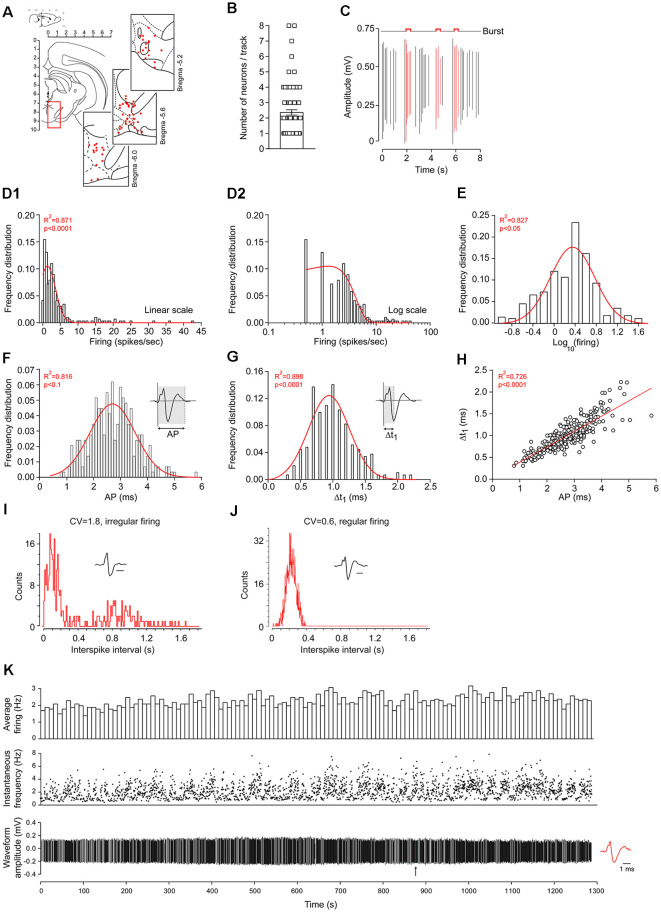
Dataset acquired using *in vivo* extracellular recordings of ventral tegmental area (VTA) neurons in anesthetized rats. **(A)** VTA location on a rat coronal brain section, adapted from Paxinos and Watson (2007). Insets: examples of coordinate locations of some neurons, reconstructed from stereotaxic measurements. All units are in mm. **(B)** Number of spontaneously-active neurons encountered during electrode descents. **(C)** Representative example of burst activities during *in vivo* extracellular electrophysiology. Red action potential (AP) waveforms are included within burst events while black AP waveforms are outside of burst events. Firing-rate distributions using linear **(D1)** or logarithmic **(D2)** scales. Firing-rate logarithmic values **(E)**. Red curves represent fitted Gaussian curves. Note that firing rates are skewed (asymmetric Gaussian, in **D1,D2**) but also lognormally-distributed (symmetric Gaussian, in **E**). Across the 291 neurons, AP **(F)** and Δt_1_ **(G)** durations were normally distributed. The direct linear relationship between AP and Δt_1_ durations **(H)**. Representative inter-spike interval distributions and AP waveforms of regularly **(I)** and irregularly **(J)** firing neurons. The horizontal bars represent 1 ms. Note the differences in coefficient of variation (CV; firing rate coefficient of variation). **(K)** Typical recording example of VTA dopaminergic (DA) neurons, displaying a stable firing rate over time. The arrow indicates the location of the AP waveform represented on the right.

Three-hundred and eleven neurons were initially recorded in 92 animals. Twenty neurons were excluded *a posteriori* due to unstable recordings. Thus, our dataset included 291 neuronal recordings in the VTA using *in vivo* extracellular electrophysiology. Positions of the recorded neurons were confirmed at the end of the experiments using stereotaxic coordinates and track positions. Examples of some recorded neuron positions are drawn in Figure 1A. Spontaneously-active neurons were recorded during electrode descents (*n* = 2.3 ± 0.2 neurons/descent; Figure 1B). Burst activity was detected using a standard protocol for DA neurons (Grace and Bunney, 1984), where at least two spikes are occurring within an interval of 80 ms or less, followed by a silence period of at least 160 ms (Figure 1C). Across our data set, firing rates were log-normally distributed (Figures 1D1,D2,E), as previously observed elsewhere in the central nervous system (Mizuseki and Buzsáki, 2013; Buzsáki and Mizuseki, 2014; Petersen and Berg, 2016). Normal distributions were also observed for AP and Δt_1_ durations (Figures 1F,G, respectively). We also observed a linear relationship between AP and Δt_1_ durations (Figure 1H). Bursting activities were not normally distributed since half of the neurons did not burst (145/291). In the remaining 146 neurons that did burst, bursting activities were not normally distributed. However, following logarithmic transformations, bursting activities presented log-normal distributions (Supplementary Figure S1). Neuronal firing regularity was assessed by calculating the CV from firing rates. Interspike interval distributions are presented as examples in Figures 1I,J. A typical recording example is provided in Figure 1K, showing stable firing rates over time.

### Hierarchical Clustering of VTA Neurons

**Figure 2:**
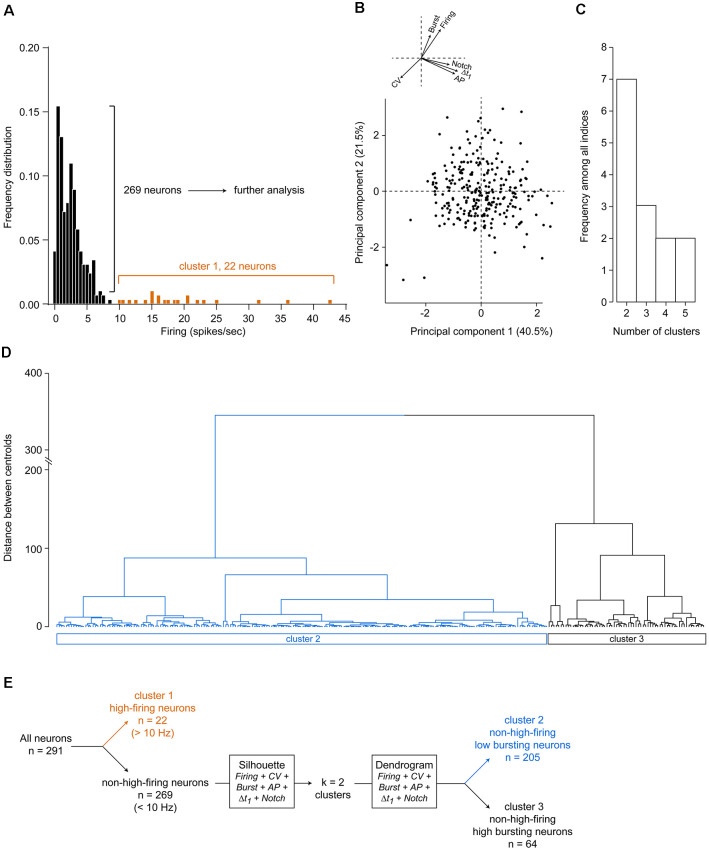
Unsupervised clustering of the 269 non-high-firing neurons. **(A)** Initial segregation of neurons with firing rates above 10 Hz. **(B)** Principal component analysis (PCA) using all six electrophysiological variables revealed that the first two principal components accounted for 62% of the total variance within the dataset. Arrows indicate vectors of the six variables. For clarity, these arrows and their respective variables are drawn again at the *top left*. **(C)** Silhouette analysis determined that the optimal number of clusters within our dataset is 2. **(D)** Unsupervised agglomerative hierarchical clustering with two clusters, using Euclidean distances and Ward’s method for optimal branching. **(E)** Methodology flow chart used in the present study.

Overall, all the electrophysiological properties were largely variable among the recorded neurons and did not allow easy distinction between non-overlapping populations when considering their distributions. To identify subpopulations of putative DA neurons, we first excluded from the dataset putative GABAergic neurons, as defined by their firing frequency above 10 Hz, which is consistently observed in the VTA (Steffensen et al., 1998; Luo et al., 2008; Li et al., 2012). In the present study, 22 neurons fired at rates over 10 Hz (Figure 2A). Hereafter, we considered these neurons as belonging to “cluster 1.”

On the remaining 269 neurons, we used several electrophysiological criteria to perform clustering within our dataset: (1) firing rate; (2) firing rate CV (firing regularity index); (3) bursting activity (i.e., the percentage of spikes in burst); (4) AP durations; (5) Δt_1_ (i.e time from the start of the depolarization to the end of the repolarization) durations as well as the (6) presence of a notched AP waveform. Using principal component analysis (PCA) on the scaled data matrix of these six electrophysiological variables, we found that 62% of total variability could be captured by the first two principal components (Figure 2B), which represents successful variance capture within the PCA analysis and adequate reduction of dimensionality. Amongst the six parameters, none could be ruled out for not inducing variance, as observed in the vectors drawn in Figure 2B. Therefore, all six parameters were subsequently used to cluster neurons. Using silhouette analysis, which determines separation distance between the resulting clusters and between each neuron, segregation of these 269 neurons was found to be efficient using two clusters, as two clusters are the most frequent ones to be output by the indices with the silhouette analysis (Figure 2C). Subsequently, hierarchical agglomerative clustering (Ward’s method) was drawn using two clusters based on these six electrophysiological properties (Figure 2D). These two clusters (“cluster 2” and “cluster 3”) segregated two different subpopulations of VTA neurons, based on optimal branching between each neuron within the entire dataset. A flow chart for the successive steps performed herein is given in Figure 2E.

### Electrophysiological Properties of Clustered VTA Neuronal Populations

**Figure 3:**
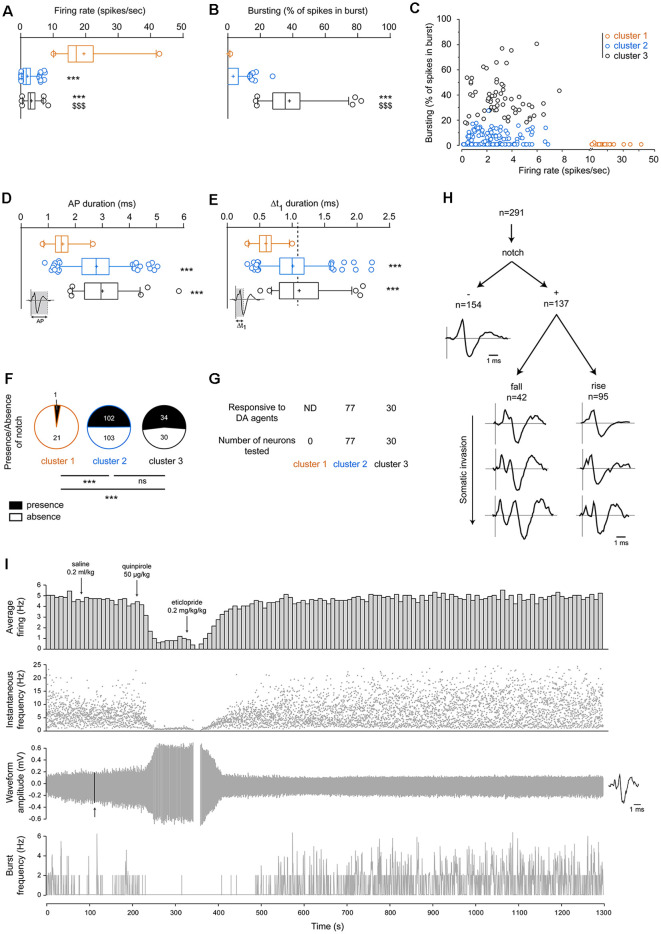
Segregation of VTA neuronal populations according to their electrophysiological properties. **(A)** Firing rates are significantly higher in cluster 1 than clusters 2 and 3. Neurons from cluster 2 displayed significantly lower firing rates than neurons from cluster 3. **(B)** Neurons from cluster 3 displayed significantly greater bursting activities than neurons from either cluster 2 or 3. **(C)** Firing rates and bursting activities in neurons from each cluster. High-firing neurons belonging to cluster 1 presented very minimal bursting activities. Generally, neurons belonging to cluster 3 (*n* = 64) presented burst activities above 17.5% of all spikes in bursts, while neurons belonging to cluster 2 (*n* = 205) displayed burst activities below 17.5%. Here, these three clusters are segregated. **(D)** Total action potential (AP) durations are significantly lower in the high-firing neurons (cluster 1) than in neurons from cluster 2 or 3. **(E)** Similarly, Δt_1_ durations from cluster 1 were significantly lower than cluster 2 or 3. The dashed line represents the well-established cut-off criterion to distinguish between DA and non-DA neurons at 1.1 ms (Ungless and Grace, 2012). **(F)** Presence/absence of notched AP waveforms in neurons belonging to clusters 1, 2, or 3. Statistical results were performed using Chi-square tests. **(G)** Neurons responsive to DA pharmacology (apomorphine 10–70 μg/kg, quinpirole 20–60 μg/kg or methylphenidate 2–4 mg/kg) were found in both cluster 2 and 3. Note that some neurons were not tested. **(H)** Within all neurons included in the present study (*n* = 291), great heterogeneity of AP waveforms was observed. One-way Kruskal–Wallis analysis of variance (ANOVA) results are given as following: ***p < 0.001 vs. cluster 1 and ^$$$^*p* < 0.001 vs. cluster 2. **(I)** Typical recording example of VTA neurons where the dopamine D_2_ receptor agonist quinpirole (50 μg/kg, *iv*) induces autoreceptor-mediated negative feedback, which is reversed by the dopamine D_2_ receptor antagonist eticlopride (0.2 mg/kg, *iv*). Note that quinpirole greatly increased the AP amplitude. The arrow indicates the location of the AP waveform represented on the right. All of the histograms represented here are box-plots (box and whiskers). The “+” sign represents the mean. The horizontal bar represents the median value. The interquartile range (Q1–Q3) is displayed with a vertical rectangle (“box”), while the thin lines (“whiskers”) represent the intervals between the lowest/highest values and the Q1/Q3 quartiles, respectively. Outlying values are represented individually with open circles but were not excluded from all analyses. Asterisks (***) are used to indicate significantly different values vs. cluster 1, while dollars ($$$) are used to indicate significantly different values vs. cluster 2. ns: non-significant.

As defined by the criteria used to isolate cluster 1 (firing rate >10 Hz), neurons from cluster 1 displayed significantly higher firing rates than neurons within cluster 2 or 3. Moreover, firing rates from neurons within cluster 2 were also significantly lower than neurons in cluster 3 (cluster 1: 19.47 ± 1.76 spikes/s, cluster 2: 2.10 ± 0.12 spikes/s, cluster 3: 3.32 ± 0.23 spikes/s, *p* < 0.001, Kruskal–Wallis one-way ANOVA, Figure 3A, Table 1). Bursting activities (defined for each neuron as the percentage of all spikes occurring in burst events) were significantly higher in neurons from cluster 3 compared to neurons from either cluster 1 or 2 (cluster 1: 0.07 ± 0.07%, cluster 2: 3.29 ± 0.36%, cluster 3: 37.97 ± 1.91%, *p* < 0.001, Kruskal–Wallis one-way ANOVA, Figure 3B, Table 1). When representing firing rates according to burst activities, we observed that neurons from each cluster formed distinguishable groups (Figure 3C).

**Table 1:** Electrophysiological characteristics of VTA neuronal populations segregated by hierarchical clustering.

	Cluster 1	Cluster 2	Cluster 3
Firing rate (Hz)	19.47 ± 1.76	2.10 ± 0.12	3.32 ± 0.23
Burst activity (%)	0.07 ± 0.07	3.29 ± 0.36	37.97 ± 1.91
Presence of bursting activity	*n* = 1/22	*n* = 81/205	*n* = 64/64
AP duration (ms)	1.54 ± 0.08	2.79 ± 0.05	3.00 ± 0.10
Δt_1_ duration (ms)	0.60 ± 0.03	1.01 ± 0.02	1.11 ± 0.05
Presence of notch	*n* = 1/22	*n* = 102/205	*n* = 34/64
Characteristics	High-firing	Non-high-firing Low/No bursting	Non-high-firing High bursting

Similarly, as expected, both AP and Δt_1_ durations were significantly shorter in neurons from cluster 1 compared to neurons from either cluster 2 or 3 [(AP durations) cluster 1: 1.54 ± 0.08 ms, cluster 2: 2.79 ± 0.05 ms, cluster 3: 3.00 ± 0.10 ms, *p* < 0.001, Kruskal–Wallis one-way ANOVA, Figure 3D and Table 1; (Δt_1_ durations) cluster 1: 0.60 ± 0.03 ms, cluster 2: 1.01 ± 0.02 ms, cluster 3: 1.11 ± 0.05 ms, *p* < 0.001, Kruskal–Wallis one-way ANOVA, Figure 3E and Table 1]. However, neurons from clusters 2 and 3 were not different for AP and Δt_1_ durations.

The presence of notch was rarely observed (1/22) in neurons belonging to cluster 1 (Figure 3F and Table 1). Remarkably, in clusters 2 and 3, half of all neurons presented notched AP waveforms (respectively 102/205 and 34/64 neurons, *p* = 0.64, chi-square test).

Some neurons (107/291), but not all, were tested for pharmacological response to dopamine D_2_ autoreceptor activation, by intravenously injecting apomorphine (20–70 μg/kg, *n* = 15), quinpirole (20 μg/kg, *n* = 26) or methylphenidate (2–4 mg/kg, *n* = 66). These neurons were found to be clustered in both clusters 2 and 3 (Figure 3G).

It is also worth noticing that, amongst all neurons, AP waveform heterogeneity was high (Figure 3H), as observed and explained by others (López-Jury et al., 2018). Indeed, notch amplitude and position occurrence (during the rise or fall of the AP) varied largely across all neurons. This indicates that the presence of a notched AP might not be an optimal discriminating factor. A typical pharmacological recording is illustrated in Figure 3I.

## Discussion

As for several brain regions, it is now clear that the VTA contains subpopulations of DA neurons. Thus, it remains crucial to differentiate these neuronal subpopulations when studying VTA neural circuits. Electrophysiological studies of VTA neurons often rely on a combination of parameters to identify the neuronal population to which they belong. Such an approach can lead to bias classification, as these parameters are usually not mutually exclusive. Moreover, several characteristics may overlap in different populations, at least concerning electrophysiological properties (Margolis et al., 2006). Here, we observed that initial sorting with firing rates above 10 Hz followed by the combination of the six classical electrophysiological parameters used in the field (firing rate and its regularity, bursting activity, AP and Δt_1_ durations, presence of AP notched waveform) into an objective hierarchical clustering resulted in the segregation of VTA neurons into three groups (Figure 2), whose characteristics are summarized in Table 1. The present study also highlights the fact that burst activities can segregate two populations with high-bursting (>17.5% of spikes in bursts) from low-bursting neurons (<20% of spikes in bursts) within non-high-firing neurons.

We used a cut-off criterion at 10 Hz to separate high-firing neurons from the remaining non-high-firing neurons, according to previous studies showing that GABAergic neurons fire above 10 Hz in anesthetized (Yim and Mogenson, 1980; Steffensen et al., 1998, 2008, 2009; Stobbs et al., 2004) and freely behaving animals (Gallegos et al., 1999; Liu et al., 2012). In these neurons, we found almost no bursting activities, which is consistent to another study, using the same recording technique (Steffensen et al., 1998). Therefore, cluster 1 represents the smallest proportion of neurons (7.6%), likely GABAergic, which are characterized by very high firing rates and almost no bursting activities, together with short AP and Δt_1_ durations. Regular firing discharges were observed in these neurons, but they did not exhibit notched AP waveforms, except in one neuron (Figure 3, Table 1). However, compared to immunohistochemistry studies, we found relatively lower proportions of GABAergic neurons (7.6%), compared to what was previously described (20–30%). This can be explained by a selection bias in part of the experiments, to focus on non-high-firing neurons (rates below 10 Hz).

Within non-high-firing neurons, clusters 2 and 3 engulfed 70.4% and 22% of all recorded neurons, respectively. These neurons are characterized by firing rates between 2–3.6 Hz (Table 1). Previous studies have shown that DA neurons present a mean firing rate of 4 Hz in anesthetized (Yim and Mogenson, 1980; Sanghera et al., 1984; Trulson and Trulson, 1987; Clark and Chiodo, 1988; Marinelli and White, 2000; Brandon et al., 2003; Ungless et al., 2004; Bennett and Gronier, 2007; Luo et al., 2008; Chernoloz et al., 2009; Valenti and Grace, 2010; Valenti et al., 2012; Oosterhof et al., 2014, 2015; El Iskandrani et al., 2015) and freely behaving animals (Hyland et al., 2002; Shen, 2003; Dahan et al., 2007; Li et al., 2012). When these neurons are recorded using *ex vivo* patch-clamp electrophysiology, lower mean firing rates are reported, around 1 Hz (Federici et al., 2005), likely due to altered circuitry dynamics induced by the slicing procedure.

When focusing on bursting activities, we found in the present study that neurons belonging to cluster 3 presented higher bursting activities (38%, Table 1) compared to neurons engulfed in cluster 2 (3%, Table 1). According to previous research, bursting activities of VTA DA neurons are included within the 20–40% range in anesthetized (Clark and Chiodo, 1988; Marinelli and White, 2000; Brandon et al., 2003; Bennett and Gronier, 2007; Chernoloz et al., 2009; Valenti and Grace, 2010; Valenti et al., 2012; Oosterhof et al., 2015) and awake animals (Hyland et al., 2002; Dahan et al., 2007; Li et al., 2012), although a few studies reported different values (Marinelli and White, 2000; Shen, 2003). Interestingly, one study reported DA neurons with low bursting activities (Marinelli and White, 2000), likely reflected in cluster 2 in our study (Table 1).

In the present study, we observed that several electrophysiological properties are normally distributed when represented on logarithmic scales. Indeed, firing rates, AP and Δt_1_ durations (Figure 1) as well as bursting activities (Supplementary Figure S1) presented log-normal distributions across all neurons. Such a principle was previously observed with firing rates in spinal motor networks (Petersen and Berg, 2016) and hippocampal pyramidal neurons (Mizuseki and Buzsáki, 2013). This suggests that skewed distributions of quantitative variables are conserved across the brain region and that such processes arise from complex and robust neuronal network computation (Mizuseki and Buzsáki, 2013). These log-normal distributions are also observed outside of neuroscience (Limpert et al., 2001).

AP properties were not discriminatory factors among VTA neurons, compared to firing patterns. Indeed, a great overlap was observed in the two clusters for total AP duration and Δt_1_ duration (clusters 2 and 3, Table 1). These characteristics are consistent with what has being described for VTA DA neurons (Ungless and Grace, 2012). Besides, the presence of a notched AP waveform is observed in around half of the neurons from both groups, showing that a certain heterogeneity exits. A recent study has explained that notched events are artifact electric event, due to the parameters surrounding the recording technique (López-Jury et al., 2018). Thus, even though AP properties are widely used to identify DA neurons, these electrophysiological parameters appear weak to segregate DA subpopulations in the VTA. The present study showed that AP durations and Δt_1_ durations are directly proportional, indicating that measuring one or the other should be sufficient. Total AP durations of neurons within clusters 2 and 3 are within the 2.8–3 ms range (Table 1). Previous studies have reported AP durations of VTA DA neurons within the 2–3 ms range in anesthetized animals (Brandon et al., 2003; Luo et al., 2008; Chernoloz et al., 2009; Mejias-Aponte et al., 2015), although another study reported a 2–5 ms range during both *in vivo* and *ex vivo* recordings (Trulson and Trulson, 1987). With *ex vivo* patch-clamp electrophysiology, AP durations are often reported to greatly vary according to projection sites (Lammel et al., 2008; Margolis et al., 2008). In awake animals, a study reported AP duration over 2 ms, with a mean at 3.5 ms (Dahan et al., 2007), while another study found shorter AP duration, around 1.5 ms (Liu et al., 2012). Finally, we also outline that non-DA neurons possess similar AP duration than DA neurons, as observed previously (Ungless et al., 2004), suggesting that AP duration is an overlapping sorting criterion.

In light with a previous study showing that AP waveforms during *in vivo* extracellular electrophysiology are strongly biased by pipette positioning and neuronal morphology (López-Jury et al., 2018), we suggest that AP properties should not solely be used to determine neuronal populations. An exception, however, should be very short AP waveforms, usually with total AP duration below 1 ms, which are consistently reported as typical characteristics of GABAergic neurons using different *in vivo* techniques (Steffensen et al., 1998; Gallegos et al., 1999; Stobbs et al., 2004; Li et al., 2012; Liu et al., 2012). Notched AP waveforms are usually selected by investigators to distinguish DA from non-DA neurons during *in vivo* extracellular electrophysiology (Ungless and Grace, 2012). While this can represent bias, we also argue that such an approach would lead to the homogeneous neuronal selection, based on similar neuronal morphologies in these neurons (López-Jury et al., 2018). The hypothesis that notched AP waveforms are solely observed in DA neurons remains an open question, which should be carefully examined in future studies. However, we suggest that experimenters should not use such a criterion to distinguish DA from non-DA neurons.

In the present study, we injected DA agonists intravenously to observed neuronal responses. However, this approach was not systematically performed, as we aimed to classify VTA neurons according to electrophysiological properties only, which would be necessary for studies where pharmacological identification of VTA neurons would consist in an *a priori* alteration of neuronal properties. All of the neurons tested responded by firing rate decrease, together with decreased bursting activities (Figure 3I). These neurons were sorted in both clusters 2 and 3, likely reflecting the fact that DA neurons are engulfed in these two clusters. The pharmacological responses of VTA DA neurons to DA agents have been extensively described in previous studies using *in vivo* extracellular electrophysiology in anesthetized animals, both from our laboratory (Bennett and Gronier, 2007; Di Miceli et al., 2019) as well as many others (Aghajanian and Bunney, 1977; Sanghera et al., 1984; Trulson and Trulson, 1987; Clark and Chiodo, 1988; Ackerman et al., 1993; Marinelli and White, 2000; Brandon et al., 2003; Guiard et al., 2008; Chernoloz et al., 2009; El Mansari et al., 2010; Valenti and Grace, 2010; Panin et al., 2012; Oosterhof et al., 2014, 2015). Similar responses were observed using *ex vivo* patch-clamp electrophysiology (Federici et al., 2005; Lammel et al., 2008; Margolis et al., 2008), although some specific subpopulations of DA neurons do not respond to such a pharmacological approach (Lammel et al., 2008; Margolis et al., 2008). Such a feature is also present in awake animals (Hyland et al., 2002; Li et al., 2012). However, non-DA neurons are also responding to such a paradigm, showing that response to DA pharmacology is shared amongst DA and non-DA neurons. Indeed, putative non-DA responded to DA agonists in anesthetized (Yim and Mogenson, 1980; Luo et al., 2008) and awake animals (Kiyatkin and Rebec, 1998), as well as in brain slices (Johnson and North, 1992; Cameron et al., 1997; Margolis et al., 2006). To note, some GABAergic neurons were found to be excited following exposure to DA agonists using extracellular *in vivo* recordings in anesthetized animals (Stobbs et al., 2004; Steffensen et al., 2008).

Beside GABAergic and DA neurons, the VTA also contains a small proportion of glutamatergic and glutamatergic-dopaminergic co-expressing neurons. These neuronal populations are less described (Hnasko et al., 2012; Morales and Margolis, 2017; Root et al., 2018) and the proportion varies widely between studies, depending on their location in the VTA [from 2% to more than 50% (Yamaguchi et al., 2011)]. Thus, we cannot rule out that glutamate-expressing neurons could be present in our clusters 2 and 3. One study reported non-DA neurons (TH-negative) with firing rates below 2 Hz (Ungless et al., 2004), which could represent glutamatergic neurons. The same study, however, also observed some DA neurons (TH-positive) with firing rates below 2 Hz (Ungless et al., 2004). Identification of either DA, GABAergic or glutamatergic neurons, using electrophysiology, immunohistochemistry, RT-PCR or optogenetics, remains equivocal (Chiodo et al., 1984; Margolis et al., 2006, 2008; Luo et al., 2008; Morales and Root, 2014; Yamaguchi et al., 2015; Morales and Margolis, 2017). Heterogeneity of VTA DA neurons could also be explained by different afferent glutamatergic inputs (Margolis et al., 2005), which we could not evaluate in the present study. To precisely parallel electrophysiological properties with the identity of each neuron, challenging experiments such as juxtacellular recording with iontophoresis (Pinault, 1996) or *in vivo* patch-clamp recordings followed by single-cell RT-PCR would be needed.

Our two groups, clusters 2 and 3, likely reflect the wide distribution of bursting activities found in DA neurons. Indeed, when identifying DA neurons, the criteria for determining bursting activities remain vague (Grace and Bunney, 1984; Ungless and Grace, 2012). Hence, our study highlights the possible relevance of bursting activity in segregating DA subpopulations in the VTA. We acknowledge that a few parameters, such as anesthesia, can alter the electrophysiological properties of VTA DA neurons, as demonstrated by previous studies (Appel et al., 2006; Marinelli and McCutcheon, 2014). Indeed, ketamine-based anesthetics are known to alter NMDA neurotransmission (Moghaddam et al., 1997) and thus the firing properties of several types of neurons (Björkholm et al., 2015; El Iskandrani et al., 2015). On the other hand, urethane can alter respiratory parameters in rodents (Hughes et al., 1982), although we observed similar electrophysiological properties between the present study and another study obtained in urethane-anesthetized animals. To control for possible electrophysiological variability, all experiments in the present study were performed under similar conditions, using chloral hydrate. Besides, numerous studies used such an anesthetic to describe the electrophysiological properties of VTA DA neurons. Comparisons between different studies, however, should be used with caution, as a few electrophysiological properties of VTA DA neurons can vary according to the recording techniques used (Sanghera et al., 1984).

To conclude, we confirm in the present study that the VTA harbors heterogeneous neuronal populations, based on hierarchical clustering analysis, at least concerning electrophysiological properties. This work highlighted the fact that firing pattern analyses cluster different subclasses of DA neurons, while AP characteristics (width and shape) are less likely to reflect such segregation. The clustering of neurons in the present study was based on electrophysiological properties, which can reflect differential excitability depending on the different inputs/outputs functions of the neuronal subpopulations found within the VTA. Finally, we suggest that only short AP duration should be used to rule out non-DA neurons within the VTA since such a trait appears as characteristic of GABAergic neurons, as well as firing rates above 10 Hz. Moreover, experimenters should not discard low-bursting neurons.

## Data Availability Statement

The datasets generated for this study are available on request to the corresponding author.

## Ethics Statement

The animal study was reviewed and approved by De Montfort University.

## Author Contributions

MD, ZH, and BG: experimental design. MD and BG: data acquisition. MD, ZH, PR, and CB-B: data analysis. MD, ZH, CB-B, and XF: data curation. DC, SL, and BG: supervisions. DC, SL, XF, and BG: funding. MD and ZH: manuscript drafting and writing. All authors: critical analysis.

## Conflict of Interest

The authors declare that the research was conducted in the absence of any commercial or financial relationships that could be construed as a potential conflict of interest.

## Supplementary Material

FIGURE S1 Bursting events across all neurons. **(A)** Frequency of bursting activities in all neurons from the dataset. **(B)** Frequency of bursting activities in bursting neurons only. **(C)** Bursting activities logarithmic values in bursting neurons.

## Abbreviations

AP: action potential
PCA: principal component analysis
VTA: ventral tegmental area
CV: coefficient of variation
DA: dopaminergic.
