# Supplementary figures and images for: In silico Hierarchical Clustering of Neuronal Populations in the Rat Ventral Tegmental Area Based on Extracellular Electrophysiological Properties
Source: Front Neural Circuits. 2020 Aug 13;14:51. doi: 10.3389/fncir.2020.00051 (PMC7438989; doi:10.3389/fncir.2020.00051)

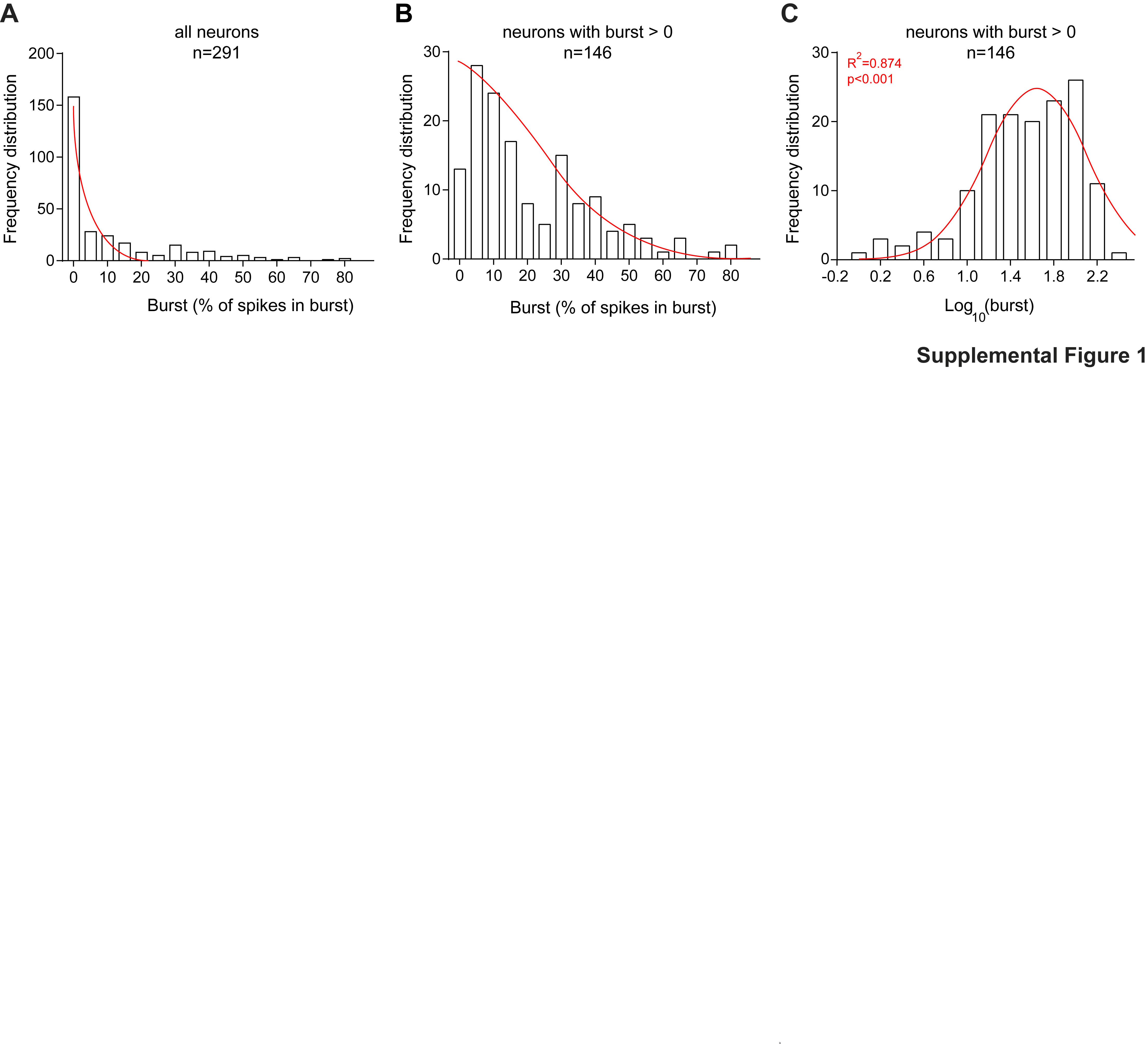

Supplement: FIGURE S1 — Bursting events across all neurons. (A) Frequency of bursting activities in all neurons from the dataset. (B) Frequency of bursting activities in bursting neurons only. (C) Bursting activities logarithmic values in bursting neurons. [file Image_1.JPEG]
